# Interplay between Competing and Coexisting Policy Regimens within Supply Chain Configurations

**DOI:** 10.1111/poms.13553

**Published:** 2021-09-06

**Authors:** Jagjit Singh Srai, Nitin Joglekar, Naoum Tsolakis, Sandeep Kapur

**Affiliations:** ^1^ Centre for International Manufacturing Institute for Manufacturing (IfM) Department of Engineering School of Technology University of Cambridge CB3 0FS Cambridge United Kingdom; ^2^ Questrom School of Business Boston University Boston Massachusetts USA; ^3^ School of Business Studies Punjab Agricultural University Punjab India

**Keywords:** bargaining power, competing and coexisting policy regimens, equity, scenario planning, supply chain netting and pooling, supply network configuration

## Abstract

Competing and coexisting policies (CACPs) may arise from the incompatibility of incentives, standards, and regulatory models between a local state and a federal government, or between two government jurisdictions across which supply networks operate. Traditional studies of supply chain dynamics typically explore the impact of policy regimens as standalone instruments. This study explores how the interplay between CACP regimens can affect the supply dynamics between producers, customers, and their intermediaries. We use a supply network configuration lens to assess implications for supply chain actors and system‐level outcomes. Our work is motivated by the federal‐state dissonance in the current dispute between India's farmers and the federal government regarding new laws that impact agricultural supply chains in India. In this case, alternative and coexisting policy interventions, ostensibly aimed at modernizing and transforming production and distribution, can lead to significant supply chain netting and inventory pooling reconfigurations in terms of material, information, and financial flows among Indian agricultural stakeholders, along with inventory repositioning and market creation options. In addition, of significance is the consequent shift in the balance between state/nation and federal/supranational equity and bargaining power, an increasingly relevant context where supply chains operate across a common but multi‐jurisdictional territory, and implications for system‐level outcomes, in this particular case equity, welfare economics, and food security. We conclude by pointing to the implications of CACP regimens, and their interplay, for the broader field of operations management and supply chain research.

## 1 Introduction

The coevolution of industrial policy and supply chain development has been a growing research agenda within the operations management (OM) community, most evident in emerging technology industries and studies supporting economic development and/or regeneration (Joglekar et al. 2016, Spring et al. 2017). Extant research has typically focused on the impact of a single policy intervention on industry structure and supply chain dynamics. This study, however, focuses on the competing and coexisting policy (CACP) context, an area yet to be addressed in the OM literature. *Coexistence* in this study refers to two separate policies (e.g., state and federal) simultaneously applied to an industry, or a component of one, such as a supply chain. *Competing* in this context indicates that coexisting policies preferentially impact diverse stakeholders ultimately leading to rivalry and performance trade‐offs. This is a significant gap in the OM literature as CACPs are increasingly common in both federal structures where devolved powers result in coexisting regional and national policies, and in international supply chains that operate across multiple jurisdictions such as across the EU post‐Brexit. This CACP context introduces additional complexity in evaluating policy intervention impacts on supply network configurations, as coexisting policies are often formulated independently with a singular focus, without consideration of the interplay between initiatives. Indeed, policy initiatives often seek to support specific stakeholders, nurture upstream supplier or downstream market developments, and/or drive system‐level benefits. Examining CACP contexts requires understanding supply chain actor behaviors, shifts in equitable outcomes and bargaining power, efficiency gains from scale, and how alternative scenarios may develop.

Industrial contexts where CACPs are evident include agriculture, renewable energy, and pharmaceuticals, where different incentives, standards, and/or regulatory models operate (see Table 1). Within federal systems, policy tensions often arise when state institutions challenge policies promulgated by federal agencies (Napolio and Peterson 2021). Of particular concern to policy makers are implications on equity (Bertsimas et al., 2012) and shifts in the bargaining power of supply chain actors (Crook and Combs, 2007), which are particularly difficult to assess within a CACP context. Examples that typify underlying CACP tensions include: (i) the U.S. Federal Power Act that exposed tensions regarding participation in interstate electricity and natural gas markets, exemplified in the case of Texas (Klump, 2017); (ii) the Brexit Referendum outcome that resulted in contentious and intensive policy development over common jurisdictional areas, such as fishing rights, food standards, and N. Ireland cross‐border protocols, requiring extensive scenario planning on various supply network reconfiguration responses (Phadnis and Joglekar, 2021) to regulatory changes and new trade protocols; and (iii) the decriminalization of the personal use of marijuana for recreation purposes in several US states such as Washington and Colorado, as opposed to federal‐based legal prohibitions which also create financial ripple effects as marijuana businesses are not allowed to access many standard banking and financial services (Carnevale et al., 2017).

**Table 1:** Competing and Coexisting Policy Paradigms Across the Globe

Sector	Description	Study/Evidence
▪ Agriculture	The U.S. Farm Bill of 2018 regulates agriculture at a federal level thus limiting potential state transformational pathways towards sustainability. The effectiveness of such top‐down federal‐level agricultural policy making has been questioned, with calls for devolution, decentralization of funding and control including the national government's ceding of some laws, policy, and programs to the regional, provincial, state, or local level.	Gundersen et al. (2004), Spangler et al. (2020)
▪ Aviation	The constitution of the US Transportation Security Administration (TSA) and the TSA's introduction of stricter protocols for passenger screening at all US airports in 2001 have created tensions with local states. In particular, local state jurisdictional responses were introduced in Texas in support of local citizen preferences for balancing security and civil liberties, as represented by the 2011 U.S. House of Representatives bills HB 1937 and HB 1938.	Ellis and McDaniel (2013)
▪ Bio‐pharmaceuticals	Custom clearance of research materials for biomedical innovation and analytical use is challenging for biomedical firms in China owing to bureaucratic procedures. China is exploring a new pilot initiative with the drug maker Merck that allows the processing of shipments with fewer application and technical dossier requirements thus improving the flow of components within biomedical supply chains that cross international borders.	Merck (2020)
▪ Pharmaceuticals	The US pharmaceuticals manufacturing industry has been outsourced during the last two decades, mainly to China and India, due to cost factors and the availability of raw materials, among other reasons. Recent developments such as the Covid‐19 pandemic highlighted a dependency on essential medicines, with calls to “reshore” pharmaceuticals production in the United States. This has triggered competing policy responses by both the United States and China to on‐shore pharmaceuticals manufacturing.	Ferry (2020), Wiley‐Law (2020)
▪ Recreational Drugs	Sale of recreational marijuana is legalized in 11 states across the United States, that is, Alaska, California, Colorado, Illinois, Maine, Massachusetts, Michigan, Nevada, Oregon, Vermont, and Washington. However, the federal government classifies marijuana as a Schedule 1 drug, which denotes no medical value and high potential for abuse. Therefore, marijuana businesses encounter challenges in accessing many standard banking and financial services.	Carnevale et al. (2017), Lenk et al. (2021)

While extensive research has evaluated particular impacts of (single‐) policy initiatives, the CACP context offers novel opportunities for research, both in terms of content and methods. In this study, we demonstrate several dimensions for novel contributions, such as the intended or counter‐intuitive changes in equity between actors, shifts in bargaining power, and system impacts. The section on future research explores further areas where the interplay between policies that are competing and coexisting provides a rich context for follow‐on research.

Given the scope of this research, we have taken a systems perspective to frame our arguments. The system boundary in our analysis includes multiple actors and decision‐makers, with multiple incentives driving different behaviors, with significant shifts in equity and bargaining power (Lee and Tang, 2018), and configuration changes to the flow of materials, information, and revenues (Srai and Gregory, 2008). Based on the equity theory, subjects should receive remuneration according to their contribution in the production of a joint endowment whereas the bargaining power denotes the magnitude of influence during an argumentation process (Rodriguez‐Lara, 2016). We have selected a detailed case study of a multi‐tier supply chain—Indian agriculture—that represents this novel CACP context, where the implications of CACP interventions at the producer (farmer), market (large retail), and system (welfare economics and food security) levels have been met with much contention. A recent federal policy initiative, the Indian Agriculture Acts approved by the Parliament of India in September 2020 (Chand, 2020), has resulted in a highly contentious public debate, legal disputes, and public protest from India's farmers and trade unions, on anticipated changes to the equity and bargaining power of farmers, and from sector specialists on supply chain implications and India's long‐term welfare economics and food security. In this study, equity and bargaining power are related but separate constructs, with the production of the joint endowment (e.g., agricultural produce) preceding the bargaining phase. In contrast, state policy initiatives promoting farmer collectivization and digital platforms aim to build scale at the producer end of the supply chain. This case study therefore provides an ideal context for examining how OM researchers might evaluate this increasingly common but underexplored CACP context. We follow the group model building method, grounded in the system dynamics literature (Hovmand et al., 2012, Vennix, 1996), in assembling system‐wide data and in validating our case inferences. Of particular interest is how, within the CACP context, we can evaluate impacts on the equity and bargaining power of producers, intermediaries, and the interests of large retailers, along with welfare economics and food security.

The dynamic operations environment, in which the CACP context applies, implies that within the supply chain boundaries executives have to consider scenario planning for outlining actionable paths to respond to emerging mandates (Joglekar and Phadnis, 2021). In the CACP context of the investigated case study, scenario planning denotes the potentially different ways in which the Indian agricultural system might evolve. The key takeaways that emerge from this case study—where the only possible explanations for the observed scenarios are associated with CACP, include:

- Alternative Supply Chain Configuration Assessments (discussed in Section 3)—We start with a current‐state conventional context where state and federal policies are aligned (i.e., neither competing nor leading to trade‐offs), to a future‐state context where these policies are not. The CACP tension yields the possibility of: (i) new types of fiscal flows, with the possibility of multiple double marginalization challenges emanating from different actors (including state institutions, federal government, and banks); (ii) novel types of inventory‐holding options created via the market; and (iii) novel types of information netting, pooling, and forecasting possibilities based on emergent market structures.
- Shifts in Equity and Bargaining Power Outcomes (discussed in Section 4)—Alternative supply chain configurations lead to changes in equity and bargaining power. Incentive incompatibility (on both the demand and supply side) will result in policy tensions. In terms of learning loops, different actors focus on different outcome metrics (e.g., federal government pursues large‐retail investments, and an enhanced food security role for the private sector, state governments focus on increasing employment and growth, and farmers seek risk‐free cash flow as well as a fair share of profits). Given the possibilities for multiple double marginalization circumstances, unique maxima may exist in such systems. The current‐state equilibrium, while far from a sustainable operating model, represents a maxima based on current regimens, albeit arrived at through a tortuous set of adjustments over many decades.
- CACP Context Understanding (discussed in Sections 3 and 5)—Method development on recognizing and understanding the CACP context enables more informed policy instrument development, alignment of policies across common jurisdictions, and can propel development of new technology‐driven market mechanisms (e.g., creation of hierarchical digital platforms). Novel market mechanisms may also yield new types of products and services.

Finally, Section 5 extends our arguments beyond the example case of India's agricultural sector, and presents research and methodological implications, spelling out a forward‐looking OM research agenda on the interplay between CACP regimens from a supply network configuration perspective.

## 2 Interplay Between Competing Policies and Network Configurations

The impact of governmental policies on supply network configurations has many dimensions depending on the nature of the interventions. In the case of public procurement of goods and services, policies need to promote positive social and economic benefits for the communities they serve (Wontner et al., 2020). The influence of particular stakeholders may also involve transaction costs related to lobbying or political considerations (Dixit, 1996) and the eponymous “power elites” (Mills, 1956). Uncertainty about trade tariff policies may motivate firms to rethink their global supply chain strategies and redirect accordingly global product flows (Dong and Kouvelis, 2020), or build in contingency based on alterative scenarios (Phadnis and Joglekar, 2021). The reverse is also the case, in that supply chain reconfiguration, for example, due to changes in product‐, production‐ or infrastructure‐related technologies, may drive regulatory change. Digitalization in particular has driven significant changes in production scale, network effects (through digital platforms), and data flows between supply chain actors requiring policy or market power, data sharing, user privacy, and governance. Ultimately, policies and regulations need to ensure that no asymmetries emerge in the distribution of the emanating benefits, particularly for the most vulnerable actors in a system (Devalkar et al., 2018). However, there is no research on the dual/multiple policy context from a supply chain perspective and the tensions that inevitably arise from competing objectives.

**Figure 1:**
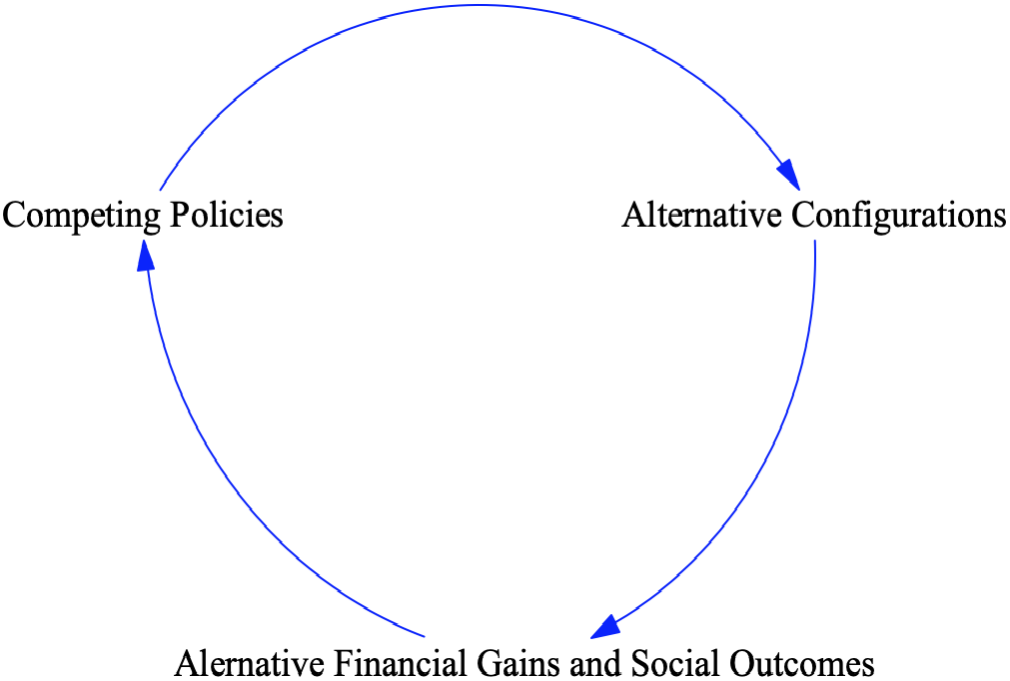
Dependencies across Competing and Coexisting Policies, Configurations, and Outcomes [Color figure can be viewed at wileyonlinelibrary.com]

In addressing this gap, we adopt a generalized system approach (Roth et al., 2016). We consider how CACPs differentially impact supply network actors and institutional players, in terms of equity and bargaining power shifts, behavior changes, individual stakeholder gains/losses, and system‐level outcomes. As alternative configurations inevitably emerge, be they policy, technology, or market driven, these in turn trigger a fresh round of policy reforms in a dynamic feedback loop (Figure 1).

**Figure 2:**
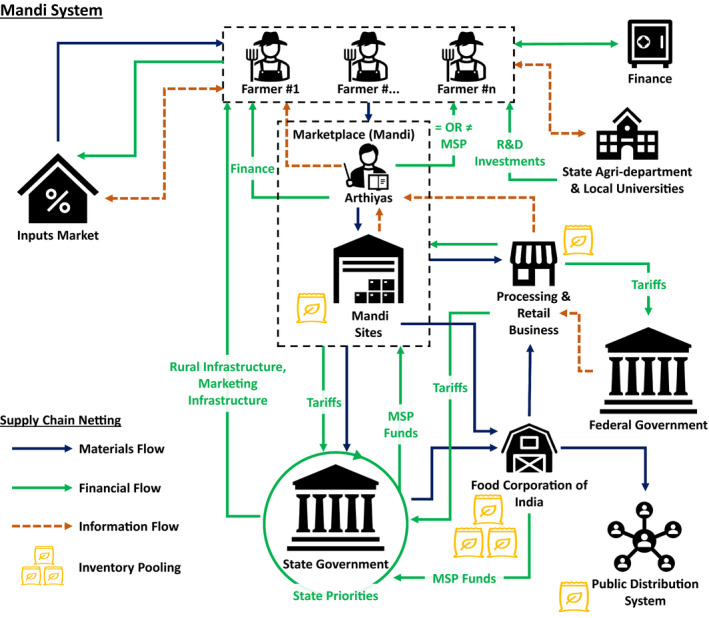
Current Supply Netting and Pooling Configuration in Indian Agriculture: Mandi System [Color figure can be viewed at wileyonlinelibrary.com]

**Figure 3:**
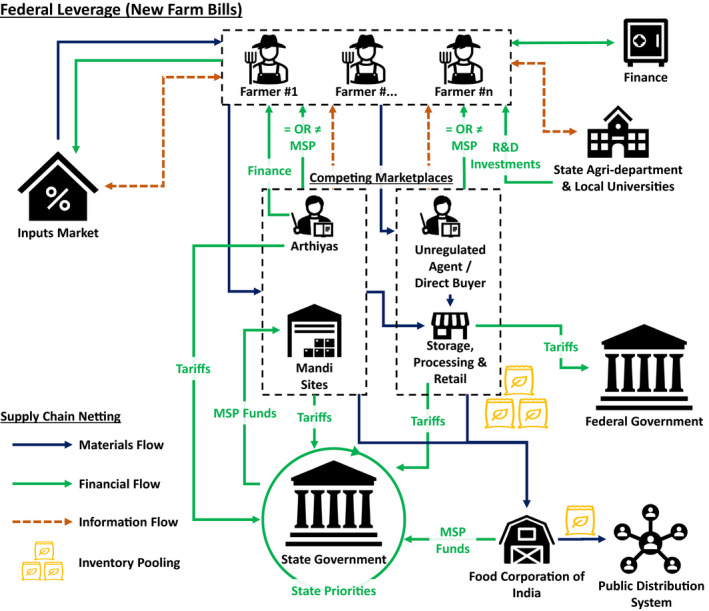
Emerging Supply Netting and Pooling Configuration in Indian Agriculture: Federal Leverage Resulting from the New Farm Bills [Color figure can be viewed at wileyonlinelibrary.com]

**Figure 4:**
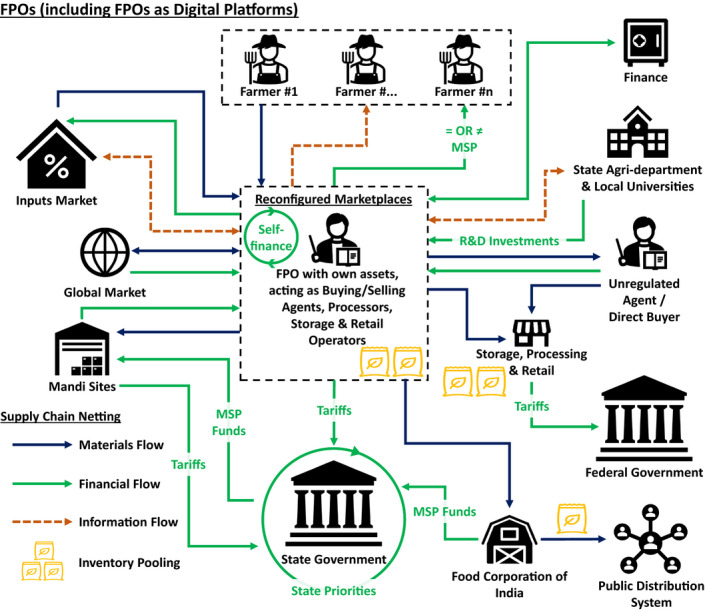
Alternative Supply Netting and Pooling Configuration in Indian Agriculture: Farmer Producer Organizations (FPOs) Including those Operating Through Digital Platforms [Color figure can be viewed at wileyonlinelibrary.com]

In considering the impact of competing policies on supply network configurations, we regard each policy as a defined scenario, which we then extend to the dual/multiple (i.e., CACP) policy context. In undertaking this analysis, we use a supply chain netting approach (Hofmann, 2007). *Netting* is defined as counting mutual payments and only paying each supply chain entity the balance. This creates cleaner information flows, and it can reduce cash holdings, transaction costs, and variable costs. We extend netting beyond its normal use in supply planning and forecasting, to the network configuration. The approach neatly identifies net changes in material, information, and revenue flows, which in our case are associated with both policy interventions and demand fluctuations. As set out in the following section, the approach has enabled us to capture configuration changes and evolving marketplaces from the baseline (i.e., Mandi) system (Figure 2), the reconfiguration prompted by India's new farm laws (Figure 3) where current and new policies compete and coexist, and the competing farmer producer organization (FPO) model proposed by the state government of Punjab (Figure 4).

These alternative network configuration diagrams identify changes in the flows of materials, financials (revenue, finance, and debt), and information. We further consider changes to physical inventory and inventory pooling models (Eppen and Schrage, 1981) in order to understand emerging consolidation points and their potential impact on bargaining power of supply network actors and on system dynamics. Recognizing the policy and welfare context of this study, particularly in terms of equity (e.g., fairness in bargaining and welfare economics among parties) (Bertsimas et al., 2012), the analysis captures the social impact of policies on stakeholders and system‐level outcomes, in our case food security, equity, and welfare economics of key system actors.

The application of supply netting, pooling, and systems thinking principles used in the study and discussed in the following section enables us to look at the system from multiple perspectives (Cheng et al., 2012) to inform our approach and to better understand this novel CACP context. These netting and pooling diagrams (Figures 2, 3 and 4) were developed by drawing on the literature in the first instance, then refined through expert interviews and workshop engagements to ensure content validity of our study's constructs. Expert inputs were drawn from policy makers, including the architects/signatories of the policy instruments, heads of FPO organizations, state agriculture administrators, finance bodies (e.g., state banks), agriculture, and supply chain experts from the Punjab Agricultural University, with multiple rounds of engagement undertaken under the auspices of the acknowledged TIGR^2^ESS research project. In this regard, the overall action research‐based method (Anand and Gray, 2017) and the subsequent analysis were revelatory to the research team and to the engaged experts in terms of system interactions. Owing to the empirical nature of the real‐world phenomena being studied, the iterative group model building process (Vennix 1996, Wacker, 1998) deployed while developing netting and pooling diagrams provided confidence in the co‐creation of supply network configuration maps and insights. Analysis on system‐level impacts required an understanding of the cause–effect relationships of each agricultural system, and the adoption of system dynamics logic (Forrester, 1961, Meadows, 1980), which has been previously successfully deployed in policy design and supply chain management (Größler et al., 2008, Kim and Oh, 2005). The analysis was therefore interdisciplinary, leveraging multiple OM research methods (Kleindorfer et al., 2005, p. 490) while exploring interfaces with other disciplines (Holweg and Srai, 2013) including economics, finance, and information systems (Roth et al., 2016), relevant to the CACP context.

## 3 Policy Tensions and Emerging Configurations: India's Agricultural Landscape

Historically, agricultural policy reforms in India have required careful consideration to address the “*very uneven distribution of the gains among regions and the adverse impact on the poor classes*” (Vaidyanathan, 2000). In this regard, a mixture of local, state, and federal government policy‐making agencies is well established in India. An overview of the current‐state agricultural policy landscape in India is provided in Appendix A. However, the policy‐making levels of India's federal government and its states are considered less‐than‐conducive to the development of aligned policy frameworks (OECD, 2018).

In September 2020, significant farmer protests broke out across India in response to new agriculture reforms introduced by the federal government, ostensibly aimed to promote investments in the sector but with many farmers, economists, and the general public concerned about likely impacts of privatization and large retail (Economist, 2020). These protests have continued into the spring of 2021. At the same time, the local Punjab state government introduced policies to develop FPOs and FPO‐led market development as part of farmer‐led business development and crop diversification strategy (Department of Horticulture, 2020). To address this CACP context, we examine these policies in terms of supply netting and pooling configurations across three scenarios in the Indian agriculture system: (i) the current State‐run Mandi system; (ii) the new Agriculture Acts introduced by the Indian federal government in 2020; and (iii) the new policy instruments introduced at the Punjab state level that promote FPOs and the scaling of FPOs through digital platforms. The agricultural systems that correspond to these scenarios are depicted in Figures 2, 3, and 4, while a description of the relevant material, information, and monetary flow shifts is inserted in Appendix B. This study focuses on crops that are under the minimum support price (MSP) regime, representing the dominant produce from states where agriculture is the most significant industrial output, which also assures farmers’ (particularly smallholders) welfare in cases of market distortions and operational disruptions.

In order to capture the realistic functionality of the CACP system, in a scientific manner, while ensuring validity of the alternative policy scenario constructs, we adopted the group model building method. This method, grounded in the system dynamics literature, enables systems thinking through problem structuring, policy simulation, and decision support (Andersen et al., 2007, Hovmand et al., 2012, Vennix, 1996). The iterative group model building process we deployed enabled us to effectively engage with Indian agriculture system stakeholders in the process of conceptualizing, formulating, analyzing, and refining the resulting CACP context. More specifically, the CACP context was presented and discussed in: (i) a workshop in March 2020, organized in India within the context of project TIGR^2^ESS, attended by Punjab agriculture policy makers and state administrators, heads of FPO organizations, finance bodies, and supply chain experts and agronomists from the Punjab Agricultural University; and (ii) five interviews with agricultural experts in charge of advising pertinent policies in India. The CACP supply netting and pooling configurations were presented to the participants who were asked to scrutinize and validate the captured variables and connections. In addition, several communications of the CACP context to Indian agriculture officers and the general public were performed in order to receive further feedback and insights. This problem structuring and conducive learning process ensured the consensus among stakeholders about the CACP context and the associated policy impacts (Rouwette et al., 2002) to inform the development of balanced policy instruments, hence further contributing to the OM field.

### 3.1 Current State‐Run Mandi System

The Mandi system (Figure 2) represents a vibrant network of economic, social, and political activities that shape relations between local and national/international networks of capital and commerce, including India's farming sector— from large agribusinesses to family‐run farms (Kapur and Krishnamurthy, 2014). It is closely associated, and many argue underpinned by, the MSP scheme, a national policy mechanism aimed to provide demand security and price stability for farmers and to safeguard small‐scale farmers from exploitation. In particular, the inbound and outbound logistics associated with the Mandi system are governed by multiple regulated agents (known as Arthiyas) who act as intermediaries, simplifying transactions between upstream and downstream markets.

The Food Corporation of India purchases crops from farmers through Mandis (see Appendix B, transacted at the agreed upon MSP for those crops covered by this regime, namely wheat and paddy) for subsequent transfer through the Public Distribution System. This Mandi system thus represents a centrally orchestrated but locally distributed food production system that supports national food security. It also guarantees a significant revenue stream for local states via local levies. For example, in Punjab State, the total receivable annual Mandi levy amounts to 6% of the value of paddy (unhusked rice) and wheat produce procured by the state's farmers (∼INR3,600 crore, equivalent to ∼US$490 million), part of which is re‐invested in rural infrastructure and local R&D Institutions (Damodaran et al., 2020; Gulati, 2020). The local economy in Punjab and Haryana States also benefits from the commission fees (2.5%) of Arthiyas.

While the Mandi system is well established and has maintained national food security for several decades, its shortcomings are widely reported. In particular, it is based on centrally managed prices for commodity crops, encourages water‐intensive and low‐value wheat–rice cropping systems, and provides limited go‐to‐market choices for farmers (Upton and Fuller, 2004). It represents an established equilibrium between state and federal governing bodies, and between supply chain actors.

### 3.2 Federal Leverage Through New Farm Bills

The introduction of three inter‐related federal farm bills in September 2020 sparked major farmer protests and nationwide strikes in support of farmers (Sharma and Sharma, 2020). Detractors of these new farm bills regard them as exploitative and assert that they undermine the existing guaranteed sale, disbursements, and safeguards of the Mandi system in favor of large food businesses, logistics providers, and retail. In terms of configuration, the bills enable direct transactions between individual farmers and private stakeholders, disintermediating regulated intermediaries, eroding MSP controls, but also the built‐in safeguards (e.g., dispute resolution) of the Mandi system (Figure 3). Detractors predict negative long‐term ramifications, including the likely demise of the Mandi system altogether as retailers, no longer constrained by strict product storage limits, are able to leverage scale and drive down prices, disadvantaging small‐ and medium‐scale farmers. Advocates of the new farm bills point to likely corporate and private business led increases to infrastructure investments and the modernization of the agriculture sector.

We examine the supply network configurational changes introduced by the federal farm bills, as illustrated in Figure 3, using the supply netting and pooling approach described earlier, which suggests significant changes to the flow of materials, finance, and information, leading to an emerging commodity marketplace that repositions inventory pools away from Mandis and other institutional bodies to the private sector.

Many agricultural stakeholders anticipate that the 2020 federal farm bills will ultimately dissolve the MSP and usher in unregulated intermediaries with increased market influence. In addition, the foreseen collapse of the Mandi system resulting from these bills will leave individual small farmers exposed to binding legal contracts with potentially new liabilities and incomprehensible clauses. Supply netting and pooling analysis also suggests that the removal of market fees and levies (Mustafa, 2020) will result in significant losses to the local states due to tariff shortfalls resulting from the effective disintermediation of the state Mandis and Arthiyas. The expected privatization of the sector may also indirectly divert tariff flows from state offices to the federal government, for example in the case of secondary processing. Figure 3 depicts these reconfigurations and Table 4 summarizes the changes in equity, bargaining power, and welfare economics across both supply chain and institutional actors, and are discussed later.

### 3.3 Farmer Producer Organizations (FPOs)

**Figure A1:**
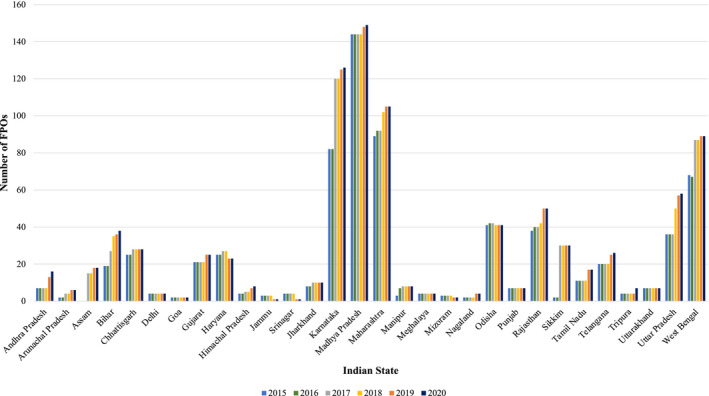
Total Number of FPOs Per Local State, 2015–2020 [Color figure can be viewed at wileyonlinelibrary.com] *Data source:* SFAC (2020).

The organizational construct of FPOs (i.e., formation of farmer groups which are registered as companies) aims to overcome the tepid performance of traditional farming cooperatives by creating autonomous business entities in competitive markets (Raju et al., 2017), each with proprietary decision‐making processes (Kuruganti, 2020). FPOs can transform marketplaces by reconfiguring supply dynamics (Figure 4), procuring inputs at commodity prices using their buying power and being producer‐owned enhancing value capture through bargaining power, scale, and direct access to markets, including supply to international commodity buyers, retailers, and customer‐centric platforms. Details about FPOs in India are provided in Appendix C. Although FPOs are a farmer‐centric mechanism that increases self‐governance and potentially the revenue of their members, it requires enhanced organizational capabilities, necessitates effective collaboration models, and introduces greater risk (and reward) into the system. However, within the major agricultural producing states, adoption rates have been rather modest (Figure A1 in Appendix C).

**Figure A2:**
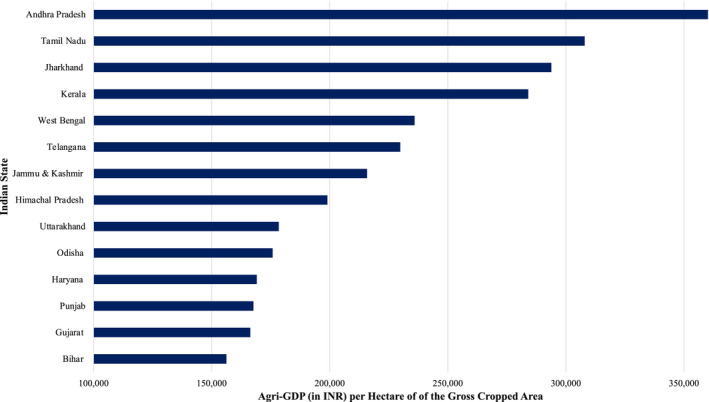
Agricultural GDP Per Hectare of the Gross Cropped Area [Color figure can be viewed at wileyonlinelibrary.com] *Data source:* Directorate of Economics and Statistics (2020).

FPOs however, have traditionally been beset with unique issues including transaction cost inefficiencies related to internal organization and governance, underdeveloped management capabilities, service quality problems, and product price variability (Bikkina et al., 2018). A Punjab state policy intervention to address these issues has also promoted “digital platforming” within FPOs, to address transparency, transactional efficiency, and build scale for overcoming operational and market challenges that currently result in modest financial returns (Figure A2 in Appendix C). Indeed, digital platforming in the agricultural sector enables producers to gain access to a wider range of customers and reduce their reliance on conventional procurement and distribution channels, and non‐value adding intermediaries in order to enhance value capture (Banker et al., 2011). Evidence from the Indian coffee sector reveals that such policy initiatives can help catalyze the efficiency of digital platforming, including: (i) reduced commodity valuation uncertainty; and (ii) increased participation of small‐scale farmers in the agricultural supply chain (Banker et al., 2011). Local institutional support for such digital policy interventions is nevertheless required, as similar initiatives, for example in distributed manufacturing models, have shown that supply network reconfigurations stimulated by advanced manufacturing and digital technologies require value provisioning investments in technological infrastructure, local institutions, and human capital (Srai et al., 2020).

Digital platforms can trigger shifts in the relations among upstream and downstream actors in manufacturing and distribution channels. From a socio‐political and economic viewpoint, the “platformization” of FPOs through digital technologies can provide regional and international scale, enhanced governance and transparency, and the free exchange of best practices as firms can leverage combined user capabilities and market processes. Of note, digital platforming of FPOs is, in principle, different from e‐procurement networks that maintain the role of a private orchestrator as a market intermediary, as in the case of ITC Limited and the e‐Choupal project (Anupindi and Sivakumar, 2006, Upton and Fuller, 2004). In FPOs that have adopted digital platforms, while intermediaries are excluded from the operations network, the power balance between them and retailers will be determined, in part, by the policy objectives set in e‐business regulatory frameworks. Similarly, the impact on income flowing to the state and federal governments can be influenced by the regulatory and policy landscape.

### 3.4 Supply Chain Netting and Pooling Configuration Summary

While Figure 4 summarizes the FPO model as a digital platform, it also exemplifies the CACP context with the interactions between the current Mandi system and the new federal farm bills. To identify configuration changes stemming from policy interventions, Table 2 summarizes the results from the supply chain netting and pooling analysis. Specifically, attributes of alternative network configurations are set out in terms of the main changes in material and financial flows for the three policy‐driven scenarios discussed. In each scenario we also identify changes in forecasting strategies, and their implications on inventory pooling locations, equity, and bargaining power, vital in understanding changes in supply dynamics and consequent provisions for food security.

**Table 2:** Emerging Types of Supply Chain Netting and Pooling Configuration Scenarios, Prompted by CACP Regimens in Indian Agriculture

		Mandi system	Federal leverage	Farmer producer organizations (FPOs)	FPOs as digital platforms
**Overall Configuration Attributes**		▪ Individual farmers cannot engage in inter‐state or international market operations.	▪ Individual farmers cannot readily engage in inter‐state or international market operations.	▪ FPOs rarely engage in inter‐state or international market operations.	▪ FPOs can easily engage in inter‐state and international market operations due to digital platforming.
	**Material Flows**	▪ Material flows are sequential following multiple intermediate transaction stages.	▪ Material flows are sequential following limited intermediate transaction stages.	▪ Material flows are direct following one intermediate transaction stage.	▪ Material flows are direct with no intermediate transaction stages.
	**Financial Flows with Netting**	▪ Financial returns based on MSP regime for wheat and paddy ➞ Spot market prices for non‐MSP crops. Selling price can be below the MSP (where MSP not implemented).	▪ Contractual arrangements for providing farming outputs— Individual negotiations between farmers & buyers ➞ Long‐term uncertainty on MSP regime.	▪ Profit margins are reasonable as FPOs can make contractual arrangements for selling farming outputs. Collective negotiations between FPOs and farming outputs’ buyers.	▪ Profit margins are large due to the scale effect and real‐time downstream market visibility.
	**Information Flows with Netting**	▪ No information about upstream and downstream markets (e.g., quality, prices).	▪ No information about upstream and downstream markets (e.g., quality, prices).	▪ Delayed upstream and downstream market information (e.g., demand, quality, prices).	▪ Real‐time upstream and downstream market information (e.g., demand, quality, prices).
	**Inventory Pooling**	▪ Inventory management is centralized by the federal government.	▪ Inventory management is semi‐centralized by retailers.	▪ Inventory management is decentralized by multiple stakeholders.	▪ Inventory management is decentralized by multiple stakeholders.

*Symbol key*: FPO – Farmer Producer Organization; MSP – Minimum Support Price.

## 4 Shifts in Equity and Bargaining Power Resulting from CACP

We now consider how policy‐driven changes to supply network configurations influence systems dynamics along with the equity and bargaining power of key actors within our CACP context. This research recognizes the importance of policy making for enabling shared value across the multiple production system actors, both in time and space. To this end, agricultural systems provide fertile ground for exploring the intersection between OM and system dynamics (Sterman et al., 2015), as they exhibit multiple feedback loops, time delays, nonlinearities, and accumulation processes within their supply networks.

The system dynamics perspective (Roth et al., 2016) deployed here considers each policy intervention at a conceptual level, including the impact of new actors on the dynamics of the system. We use this approach to evaluate potential policy impacts for each scenario, and in the combined CACP context, including repetitive policy‐verification cycles and feedback loops (anticipated or not) to inform effective policy interventions. Furthermore, the adoption of the Transaction Cost Economics Theory (Williamson, 1979, 1993) has been effectively deployed by OM and supply chain scholars (e.g., Ketokivi and Mahoney, 2020) to study policy implications and changing system's dynamics.

This section also explores the relationship between configuration and bargaining power, extensively discussed in the literature (e.g., Li and Amini, 2012), and how shifts in bargaining power ultimately impact an actor's financial performance (Lanier et al., 2010). The novelty here is in the unexplored CACP context, and how the system dynamics approach can shed light on the likely impact of policy interventions on key actors.

### 4.1 Equity and Bargaining Power Attributes

**Figure 5:**
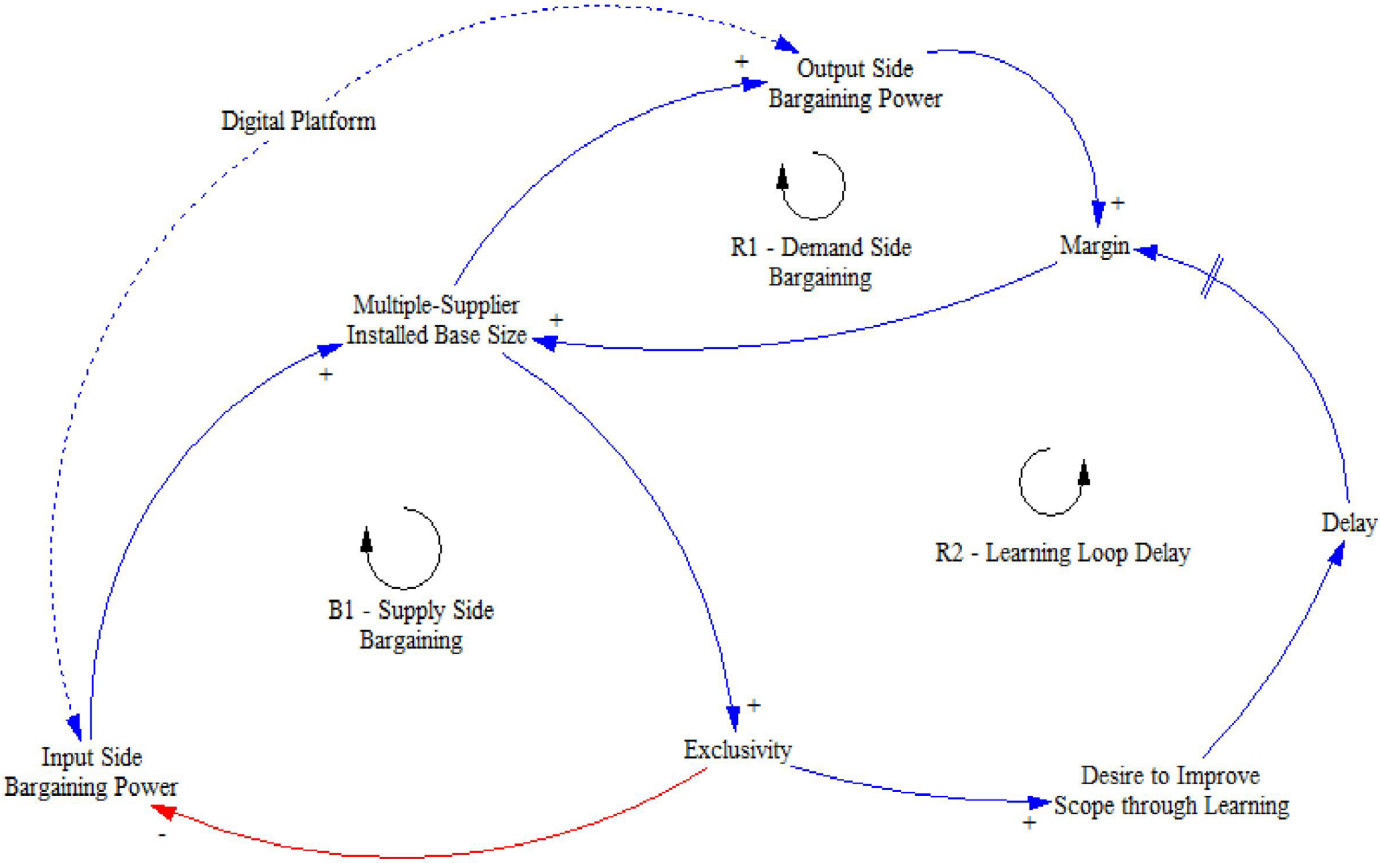
Extended Linkages Within a Supply Network System [Color figure can be viewed at wileyonlinelibrary.com]

From a system design perspective, this research explores how CACP may drive economies of scale and changes to equity and bargaining power. First, FPOs attain increased bargaining power in upstream food supply chain transactions (e.g., procurement of farming inputs such as seeds, fertilizers, fuels), providing farmers with new mechanisms to effectively negotiate with suppliers on enhanced terms of trade and prices (Michalek et al., 2018) (Balancing Loop, B1—Figure 5), thereby improving equity opportunities. Second, the increased bargaining power of farmers can ensure the direct and remunerative interaction with downstream network stakeholders, leading to market price mark‐ups compared to field‐gate prices (Trebbin, 2014) (Reinforcing Loop, R1—Figure 5), hence improving opportunities for equity at scale. Third, digital platforms enable enhanced data management and information visibility, leading to flexibility, agility and robustness against operational disturbances (Veeramani et al., 1993) (Reinforcing Loop, R2—Figure 5). Finally, realizing bargaining power balance across agri‐food supply chains can catalyze entrepreneurial opportunities across a range of operations (e.g., financing, processing, and marketing) and generate shared value for farmers (Tang, 2018).

Figure 5 captures two reinforcing loops and one balancing loop between demand‐side and supply‐side bargaining and learning. Combining these loops prompts the trade‐off dynamics and interplay between the system elements across the “Supply‐side Bargaining,” “Demand‐side Bargaining,” and “Learning Loop Delay” loops. Table 3 illustrates the equity, bargaining power, and learning loops’ attributes for the different CACP scenarios pertinent to our case study.

**Table 3:** Equity and Bargaining Power Attributes for Supply Chain Configuration Scenarios Resulting from CACP in Indian Agriculture

	Mandi system	Federal leverage	Farmer producer organizations (FPOs)	FPOs as digital platforms
**Supply‐Side Bargaining and Equity**	▪ Individual farmers have limited bargaining power in their interactions with farming inputs’ suppliers due to the absence of upstream market visibility (e.g., quality, prices). ▪ This reflects the status quo and raises questions about equity.	▪ Individual farmers have limited bargaining power in their interactions with farming inputs’ suppliers due to the absence of upstream market visibility (e.g., quality, prices). ▪ This raises questions about equity, for example, for smallholders.	▪ FPOs have increased bargaining power with farming inputs’ suppliers due to scale and delayed upstream market visibility (e.g., quality, prices). ▪ This improves opportunities for equity.	▪ FPOs have significantly increased bargaining power in interactions with farming inputs’ suppliers due to scale and real‐time upstream market visibility (e.g., quality, prices). ▪ This improves opportunities for equity at scale.
**Demand‐Side Bargaining and Equity**	▪ Individual farmers have limited bargaining power in interactions with farming outputs’ buyers due to the absence of downstream market visibility (e.g., demand, quality, and prices). ▪ This reflects the status quo and raises questions about equity.	▪ Individual farmers have limited bargaining power in interactions with farming outputs’ buyers due to the absence or delayed visibility of the downstream market (e.g., demand, quality, and prices). ▪ This raises questions about equity, for example, for smallholders.	▪ FPOs have increased bargaining power in interactions with farming outputs’ buyers due to the scale effect and delayed downstream market information (e.g., demand, quality, and prices). ▪ This improves opportunities for equity.	▪ FPOs have significantly increased bargaining power in interactions with buyers due to the scale effect and real‐time downstream market visibility (e.g., demand, quality, and prices). ▪ This improves opportunities for equity at scale.
**Learning Loop Delay**	▪ The learning curve is flat. Individual farmers do not have visibility over upstream or downstream markets. ▪ No desire to improve scope due to “survival” focus.	▪ The learning curve is flat. Individual farmers do not have visibility over upstream or downstream markets. ▪ No desire to improve scope due to “survival” focus.	▪ Learning enabled via engagement in the collectivist instrument (i.e., FPO). ▪ The learning curve gradually increases, hence building trust through membership.	▪ Learning enabled via engagement in the collectivist instrument (i.e., FPO), access to digital services, and real‐time visibility to both upstream and downstream markets. ▪ The learning curve continuously increases, building trust through data transparency.

### 4.2 Changing Dynamics and Equity Profiles in India's Agriculture Supply Chain

Within the Mandi system, while farmers’ bargaining power is limited, their sales at the MSP is effectively guaranteed. Arthiyas, regulated intermediaries, have elevated bargaining power as they fulfil their responsibilities with regard to the auction and delivery of crops to and from Mandis, and often adopt additional roles as moneylenders (Singh et al., 2008).

The conceptual framing of the CACP context suggests that the new farm bills may drive a more “free market” model that could favor large private retailers and international conglomerates with enhanced bargaining power by leveraging their scale and infrastructural investments. In addition, the bargaining power of individual farmers is expected to diminish due to the removal of the price reference from essential commodities, while the invisible production costs (e.g., hidden family labor, land‐holding costs) in price setting are expected to be disregarded (Chawla, 2020).

Finally, FPOs redirect bargaining power to the farmers (Nagarajan and Bassok, 2008) and their business scope can span from seed cultivation to distribution services, disintermediating intermediaries. The advent of digital platforms can enable the small producers of FPOs to harness the benefits of network effects (Wang and Miller, 2020) and engage with international markets (Srai et al., 2020). The consequent shifts in the equity and bargaining power of supply chain actors have generated rich debate on digital platforms and their mediating role between supply and demand, but less so on the redistribution of value between producers, retailers, and customers.

The bargaining power shifts and equity change implications of these CACP regimens are summarized in Table 4. The analysis suggests that the CACP policy landscape will lead to major changes in the equity and bargaining power of both supply chain and institutional actors, with wider implications for the welfare of agricultural system actors, particularly farmers. The interplay between policy initiatives within the CACP context also suggests longer term implications at the system level on food security. The analysis, summarized in Table 4, validated with key stakeholders (i.e., policy makers, farmers, FPO leads, industry experts, and academia), through the iterative approach discussed earlier, provided confidence in the relevant considerations that are required, potential implications of the dual/multiple policy landscape, and equally important, the revelatory power of the approach itself.

**Table 4:** Changing dynamics and equity scenarios in the Indian agriculture supply chain system

Equity considerations	Mandi system	Federal leverage	Farmer producer organizations (FPOs)	**FPOs as digital platforms**
**Equity and Bargaining Power in the Supply Chain**				
▪ Farmers	⟷	↓	↑ (self‐regulated)	↑?↑ (digitally enabled)
▪ Intermediaries	↑ (regulated)	↑↑ (unregulated)	N/A (disintermediated)	N/A (disintermediated)
▪ Major Retailers	N/A	↑↑	⟷	↑?↓
**Equity and Welfare Implications**				
▪ Local Government	↑ (local state tariff income)	↓	↑?	↑↑?↓
▪ Federal Government	↓?↑ (food security)	↓?↑	↑?	↑↑?↓

*Symbol key*: “⟷” – neutral effect; “↑” – increased equity; “↓” – decreased equity; “?” – uncertain equity outcomes; “↑?↓” – optimization achieved depends on objectives and parameters that are set; N/A – Not Applicable.

## 5 Research Agenda

This study has focused on the systemic shifts in supply network configuration, equity, and bargaining power as a consequence of CACP regimens across a common but multi‐jurisdictional territory. The case example— CACP policy interventions in the Indian agricultural context— has shown that while some level of policy innovation is clearly needed, the tensions arising from competing policies cannot be evaluated using conventional (i.e., single policy) analyses. We have shown how such innovations can be evaluated using a supply network and systems lens for each alternative scenario in the context where they coexist, and importantly, we show the possible implications for the primary actors, across both the supply chain and more broadly, across relevant state and federal bodies. Such tensions between simultaneous policy regimens are also evident in multiple settings, as exemplified in alternative renewable energy mandates by various US state governments (Shields, 2021), competing standards for active pharmaceutical ingredients in the United States and China (CFDA, 2021), and unique data privacy regulations in various countries within the EU (GDPR, 2021).

Going beyond the particular scenarios considered in the Indian agriculture sector, broader questions arise as to how simultaneous and differing policy regimens and their associated incentives interact with each other and the potential incompatibility of these coexisting initiatives. In the CACP context, at the state or federal government level, or between two different government jurisdictions (e.g., the United Kingdom and EU) across which a supply network may operate, frameworks are needed to evaluate the impact from an OM perspective. This research attempts to address this gap on the OM literature. As our study has shown, analysis frameworks should consider both individual policy interventions and most importantly for the real‐world context in which OM scholars operate, their interplay. This prompts a series of configurational questions around production and supply chain systems research within a competing multi‐jurisdictional policy context, an increasingly common scenario, as we consider both intra‐country (devolved regions or states within federal systems) and international supply chains. Furthermore, we have shown that policies promoting digital platforms can have scale effects, both at the supply end and market end and provide further scenarios to examine. We therefore identify an exciting agenda in this coexisting, multiple policy, and common jurisdiction context across four, related but separate, research streams:

1. Supply Chain Configuration research that explores multiple coexisting policies within a common jurisdictional area.
2. Digital Platform‐based Hierarchical Systems that provide scale within this particular policy driven supply‐market context.
3. Scenario Planning approaches to explore implications of competing (coexisting) policies and the likely impact on stakeholders.
4. Interdisciplinary Research related to the CACP context and its interface with OM.

### 5.1 Supply Chain Configuration

Extant research in integrated supply systems has to date concentrated on examining local and global optima for supply chains. In particular, the traditional focus has been on strategic inventory placement choices (Graves and Willems, 2008), while more recent studies extend the perspective over service level and inventory costs and further consider equity across delivery locations (Gallien et al., 2021). We have argued that CACP regimens can shift the focus of supply chain configurations to non‐traditional constructs, for example, the equity and bargaining power across supply system actors and their social, operational, and financial consequences. For instance, many conventional studies have looked at double marginalization issues based on the interactions between inventory decisions and information architecture for a single policy structure (Roy et al., 2019). However, coexisting policies for the same jurisdiction open up alternative possibilities for equity, bargaining, and double marginalization (e.g., based on alternative financing and tax management mechanisms).

**Figure 6:**
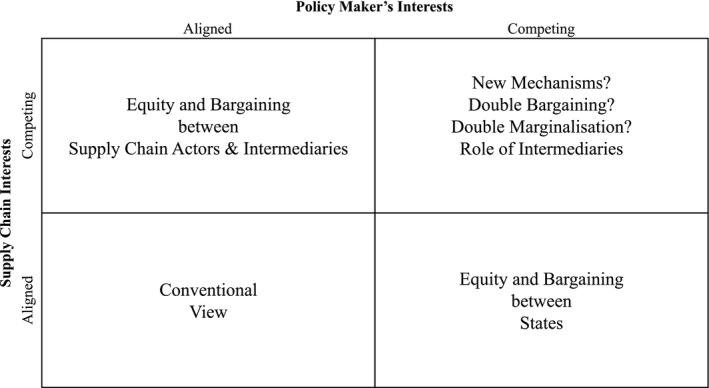
Alignment across Policy Makers’ and Supply Chain Interests

Such possibilities may lead to research on novel configurations including some studies where a segment of the supply chain is intentionally decoupled to account for competing policy structures, wherein particular segments may be optimized for specific policy regimens (e.g., separate governance for supply chains for Asia and Europe). We illustrate these research possibilities though the alignment matrix shown in Figure 6. From a configuration perspective, a series of research questions emerge. How can local supply chain systems be tuned, and selectively decoupled, to take advantage of local regulations? How do we model such systems, based on local versus global laws? Is it possible to isolate and decouple local and global metrics based on policy differences and then tune configurations accordingly? Can disintermediation be achieved while ensuring viability of operations, empowering entrepreneurial dynamics (e.g., of farmers, in our case), and also benefitting state institutional interests?

### 5.2 Platform‐Based Hierarchical Systems

Two‐sided platforms have been receiving increasing attention (Van Alstyne et al., 2016, Parker et al., 2017, Tan et al., 2020). The key configurational question here focuses on the supply network position of digital platforms. This notion of “platform centricity” (e.g., farmer–producer centric; Mandi centric; retailer centric; consumer centric) can substantially impact a supply chain system's efficiency in the distribution of equity, bargaining power and in value capture. To avoid such imbalances, we argue that it may be possible to introduce a “platform hierarchy” characterized by a multi‐level platform architecture to manage CACP regimens. Key research questions in this context are whether a central “platform of platforms” can help regulate power distribution and coordinate operations at more abstract levels of the hierarchy thus accommodating the different interests of the various supply chain actors. The exploration of the proposed notions of platform centricity and platform hierarchy addresses an evident gap in the extant literature. How do these platform notions of centricity and hierarchy impact supply chain design and the development of future digital platform business models? For example, dairy farmers can develop value‐added products such as new varieties of cheese and yogurt. Indeed, do digital platforms inevitably drive the move to product‐service models that require policy innovations related to end‐user engagement (and governance thereof)? And, from a fiscal perspective, do digital platforms create dissonance between points of value creation and value capture?

### 5.3 Scenario Planning

Due to the complexity of CACP regimens on potential shifts in equity and bargaining power, we argue that appropriate supply chain scenario planning (e.g., Joglekar and Phadnis, 2021) is vital to assess the various implications of alternative policies. To this effect, digital platform technologies can leverage public and private data and information sources to inform supply chain planning. The dynamic changes occurring in global and local business landscapes require that scenario‐centric thinking about supply chains be applied at shorter intervals (e.g., less than a year) compared to the past where planners considered a strategic horizon of 10, 20, or even 30 years. The Covid‐19 pandemic has shown that dynamic changes and unpredictable or rare events, which can have global impacts on multiple fronts, should no longer be considered as “black swans.” Specifically, the pandemic exposed the fragility of global supply chain systems and further highlighted the need for scenario planning strategies to accommodate different demand environments, mitigate supply shocks and respond to demand volatility to ensure supply chain resilience and operational continuity. Policy heterogeneity (e.g., some Covid‐19 vaccines have been approved in some countries and not others, or some of the crew in maritime supply chains have been “stranded” in different countries based on differing standards of pandemic safety rules’ compliance) and the need for rapid response can result in multiple configuration optima. Scenario analysis approaches also raise questions about alternative netting and pooling forecasts and constitute new research frontiers for the supply chain management field. Specifically, in agriculture value chains, scenario planning could further propel resiliency and eco‐friendliness (Dong, 2021).

### 5.4 Interdisciplinary CACP Research

The CACP dilemma is discussed in the macroeconomics literature; however, it is not explored in the OM field. To that end, interdisciplinary and multi‐disciplinary research opportunities exist in adjacent fields, in particular, in Decision‐Making in Public Policy & the Social Good, Development Economics, and Agricultural & Natural Resource Economics, all fertile fields to explore the CACP context. Our findings on the tensions between state and federal bodies, or international tensions based on conflicting laws and regulations, suggest that the fields of Political Economy, Political Institutions, and Federalism & Sub‐National Politics are also relevant. These research fields recognize supply chain risks, but do not disaggregate or aggregate it with precision in terms of netting and pooling possibilities. This calls for interdisciplinary and multi‐disciplinary research and the synthesis of these domains into new methodological approaches, data sources, and analytics skills. Each of these research realms typically assumes the configuration of supply chains as a given, and that theory building focuses on creation and assessment of CACP policies from a macro perspective. However, dual causality is also a possibility, such that the creation of alternative configurations may yield a new type of equilibrium within CACP structures. Moreover, OM contributions to public policy raise CACP research queries with regard to associated spillover effects and unintended consequences (Catena et al., 2020). In this vein, feedback loops dictate the need for interdisciplinarity to inform the systemic reconfiguration of value chains across all stages to balance financial, ecological, human, and socioeconomic outcomes (Roth and Zheng, 2021). For example, what are the system or stakeholder performance thresholds? To what extent would deviation from a desired state necessitate further policy interventions to encourage supply network reconfiguration, aimed at rebalancing bargaining power and consequent stakeholders’ returns?

We therefore argue that these four agendas within the novel CACP context represent exciting new areas for OM researchers and those from related domains, to explore in future research.

## Appendix A Agricultural Policy Landscape in India

This agricultural supply chain involves a multi‐tier supply network with a significant role of state actors and has seen in 2020 major policy reforms introduced by both local state and federal governments. These policy initiatives will impact the long‐established agricultural markets or market yards (Mandis) that have underpinned the supply model, representing a distributed network of collection and procurement centres, with transactions undertaken at a pre‐set Minimum Support Price (MSP). As well as securing a baseline income to individual farmers, the Mandi–MSP model has provided a critical national food system objective, namely that of food security. The federal reforms introduced in September 2020 have been highly contentious in terms of their potential impact at both individual stakeholder (farmer and local state revenues) and at the system (national food security) levels (Economist, 2020). These reforms, at face value are market‐oriented, aiming to attract private‐sector investments in supply chain infrastructure (i.e., equipment, warehousing, distribution, technology) to promote diversification to more high‐value crops and food processing, drive scale in support of modern retail, and thus reducing the dependency from intermediaries or middlemen who may exert elevated control in these wholesale markets.

In this fluid regulatory landscape, both state and federal policies have sought to encourage the development of Farmer Producer Organisations (FPOs). The FPOs are legally recognised as organisational constructs that enable the collectivisation of small, marginal and landless farmers. Similar to cooperatives, FPOs provide scale and management capability, improving substantially the bargaining power of farmers in their engagements with upstream and downstream markets and supporting the development and offering of value‐added services and products. State policy FPO development initiatives have also embraced potential opportunities that arise from the introduction of digital platforms, providing mechanisms for connecting farmers at scale thereby delivering enhanced leverage with buyers and sellers, as well as opportunities for improved coordination and services for their members, and the coordination of FPO operations.

To explore the impact of CACP, we consider the current well‐established Mandi–MSP system as being the baseline equilibrium state that has evolved over many decades. Key aspects of this current system, also referred to as the foundation of India's Green Revolution, has involved a number of policy initiatives including the consolidation of landholdings in 1953, the introduction of the MSP in 1968, and the formation of the Agricultural Produce Market Committee in 2003. These policies together with institutional investments in state agricultural universities, procurement agencies (Food Corporation of India) and a Public Distribution System have resulted in delivering national food security. This represented a careful balance between the states, where responsibility lies for agricultural policy, and the federal government accountable for national food security.

## Appendix B Supply Chain Netting and Pooling Scenarios in the Indian Agricultural System

In order to exemplify the CACP context, we investigate the real‐word case of the Indian agricultural system and we examine the competing and coexisting policies between regional and federal governments, along with implications on equity, bargaining power, and welfare economics across both supply chain and institutional actors. In terms of analytical framework, to understand the CACP context we used the supply netting approach (Hofmann, 2007) to capture related shifts in the underpinning flows (i.e., material, financial, information) and inventory pooling. The emerging types of supply chain netting and pooling configuration scenarios, prompted by CACP regimens in Indian agriculture, include: (i) the current State‐run Mandi system (status quo); (ii) the new Agriculture Acts introduced by the Indian federal government in 2020; and (iii) the new Punjab state level policy instruments that promote FPOs and the scaling of FPOs through digital platforms. Finally, to validate these scenarios we applied systems thinking principles, leveraging inputs gathered through workshop engagements and a series of interviews with Indian agricultural system stakeholders. These enabled the understanding, formulation and refinement of the resulting system interconnections (Roth et al., 2016) and the CACP context.

- *Scenario I: Mandi System – as depicted in Figure 2*

In the traditional Mandi system, individual farmers procure material inputs from the open market on the understanding that those agri‐produce designated for procurement under the Minimum Support Price (MSP), namely grains in the case of Punjab, will be procured in their entirety by government agencies, by either local state actors through Arthiyas (i.e., regulated intermediaries) or federal agencies The procurement of farming inputs to accommodate the expected demand requires upfront investment on the farmer‐end thus driving their majority (particularly marginalised and smallholder farmers) to seek external financing, often leading to excessive debt exposure. In most instances, finance is provided by Arthiyas who adjust sales of the final produce of the farmer against the finance provided and interest to be paid. Following the harvesting season of the MSP crops, farmers deliver their produce to Arthiyas who facilitate material transactions in the supplier‐buyer system and reimburse the farmer based on the quantity and quality of the delivered produce, and the MSP marketplace dynamics. Thereafter, Mandis facilitate the distribution of the agricultural produce to the state, which through the Food Corporation of India (where the magnitude of the inventory pooling resides) and the public distribution system ensures national food security. The Arthiyas’ facilitated transactions ensure significant state tariff income that supports rural infrastructure development, marketing, and research and development investments. In addition, Mandis may deliver produce to the private processing and retail business, that then attributes tariffs to both the state and federal governments based on the scale and scope of downstream transactions.

- *Scenario II: Federal Leverage (New Farm Bills) – as depicted in Figure 3*

In the federal leverage system, effectuated through the new farm bills on 2020, alternative competing marketplaces emerge with which farmers have the option to transact. First, farmers can indirectly transact to the traditional public Mandi system through the Arthiyas as these are the regulated intermediaries. Alternatively, farmers can directly engage to the private market that consists of unregulated agents and other private storage, processing, retail and individual actors, avoiding Mandi fee/tariff charges. In federal leverage system, farmers still need to procure production inputs, frequently in debt, whilst considering multiple demand signals from both official sources (i.e., government agri‐department, local universities, Arthiyas) and the emerging private market stakeholders. In the CACP context, the traditional Mandi system is provisioned to continue operating under the established market status quo. However, the private marketplace is anticipated to shift the magnitude of material transactions from the public to the private marketplace; hence, the emerging unregulated marketplace potentially becomes the inventory pooling focal point with still unexplored yet alarming repercussions to national food security and farmers’ welfare economics. To this end, the private marketplace is also expected to shift the magnitude of tariff streams from the state government to the federal administration with further implications to public investments for the overall rural development. Ultimately, marketplace dynamics are expected to influence the future MSP regime while farmers’ target market choice defines their remuneration level.

- *Scenario III: FPOs (including FPOs as Digital Platform) – as depicted in Figure 4*

In the FPO system, the marketplace is reconfigured as farmers formulate conglomerations to leverage the effects of scale economies through accumulating assets and capacity, and developing and offering an extended array of agricultural‐related products and services. In this regard, FPOs are becoming the focal point in agriculture with significant spillover effects. First, in the FPO system the procurement of farming inputs and access to financing sources is managed centrally hence safeguarding farmers from excessive debt exposure. Second, FPOs become the dominant entity to directly transact with state Mandis, the Food Corporation of India, various storage, processing and retail actors, and the unregulated agents. As the transactions are centrally coordinated and managed at scale, multiple material, financial and information flows are created that help balance food security and tariff income to state and federal governments, while farmers are safeguarded from contractual pitfalls and receive a fair remuneration. Third, the FPO system helps balance inventory pooling between state FPOs and private actors thus ensuring food security and market growth opportunities. At a greater extend, the digital platforming of FPOs could allow leveraging the value of information by enabling greater market transparency and agri‐food supply chain visibility (both upstream and downstream). Furthermore, digital platforms could allow the extroversion of farmers by facilitating transactions in the international market whilst enabling further operations opportunities and financial growth prospects.

## Appendix C Farmer Producer Organisations in India

In India where the agri‐economy sector employs about 60% of the population, some 126 million marginal and small farmers (i.e., < 2 ha of farmland) constitute the majority of cultivators (∼ 85%) (Raju et al., 2017). However, smallholder farmers, when operating individually, experience limited participation at both input supply‐side and end‐product market echelons (Sodhi and Tang, 2011), impeding the advancement of rural systems (Barrett, 2008). Farmers’ collectivisation, e.g., in the form of cooperatives, is an intrinsically appealing norm of agricultural production in emerging economies as it allows smallholders to consolidate individual outputs and harness the benefits of scale (An et al., 2015; Ma and Abdulai, 2016). To that end, the mechanism of FPOs aims to help overcome the tepid performance of traditional cooperatives by enabling farmer groups to operate as autonomous business entities in competitive markets (Raju et al., 2017). Equity‐based incentives and the organisational structure of an FPO could then encourage the participation of farmers and determine the viability of the respective operations (Li et al., 2021).

Although FPOs are a farmer‐centric mechanism that can help increase self‐governance, scale and ultimately revenue of their members, FPO growth rates are rather modest (Figure A1). Furthermore, in Punjab, the ‘breadbasket’ of India, the total number of FPOs and the number of FPO registered farmers is relatively small, thus denoting an underlining paradox. Punjab has been limited to the production of staple goods; the state produces about one‐fifth of the national wheat supplies and one‐tenth of the country's rice production. Diversification to high‐value crops like fruits and vegetables, as viable options to the dominant ‘wheat‐paddy’ system, and the involvement into other agribusiness activities have not progressed despite the impressive farming capacity and capabilities. This is reflected in the value creation numbers for Punjab; in terms of agri‐GDP per hectare of the gross cropped area, Punjab is lagging behind several other local states (Figure A2). The new Punjab state FPO policy notification (Department of Agriculture and Farmers Welfare, 2020), also issued in September 2020, aims to address current disincentives to FPO formation and development. Its development, unlike the new Indian Agriculture Acts, involved extensive discussion with local stakeholders including state policy leaders, farmer organisations, finance providers and academia (The Tribune, 2020).
